# Imaging overpressurised fracture networks and geological barriers hindering fluid migrations across a slow-deformation seismic gap

**DOI:** 10.1038/s41598-023-47104-w

**Published:** 2023-11-11

**Authors:** Ferdinando Napolitano, Simona Gabrielli, Luca De Siena, Ortensia Amoroso, Paolo Capuano

**Affiliations:** 1 Dipartimento di Fisica “E. R. Caianiello”, Università degli Studi di Salerno, Via Giovanni Paolo II, 84084 Fisciano, SA Italy; 2 Istituto Nazionale di Geofisica e Vulcanologia, Via di Vigna Murata, 605, 00143 Rome, Italy; 3 Institute of Geosciences, Johannes Gutenberg University, Mainz, Germany; 4 Dipartimento di Fisica e Astronomia “Augusto Righi”, Alma Mater Studiorum Università di Bologna, Viale Carlo Berti Pichat 8, 40127, Bologna, Italy

**Keywords:** Solid Earth sciences, Geophysics, Seismology

## Abstract

There is an ongoing debate on the processes producing background seismicity and deformation transients across seismic gaps, i.e., regions that lack historical large-magnitude earthquakes. Essential missing elements are geophysical images that resolve sources of geophysical unrest. Here, we apply seismic scattering and absorption tomography to data recorded during the 2010–2014 seismic sequence within the Mt. Pollino seismic gap region (Southern Italy). The tomographic models show high sensitivity to fluid content, deformed fractured structures, and impermeable layers stopping fluid migrations. They bridge the gaps between geological and geophysical models and provide a highly-resolved image of the source of seismic and deformation unrest within this seismic gap. High absorption topping the western Pollino seismic volume appears pressurized between the low-Vp/Vs and low-scattering San Donato metamorphic core and a deep basement. Absorbing fluids can only migrate laterally to the east, blocked in the west and southwest by deep low-scattering barriers associated with east-dipping faults and to the north and southeast by saturated overpressurized low-scattering basins. This eastern migration is only partially effective, producing seismicity across the lowest boundary of the high-absorption volume. Our results showcase the potential of seismic scattering and absorption when imaging structures causing geophysical unrest processes across fault networks.

## Introduction

**Figure 1:**
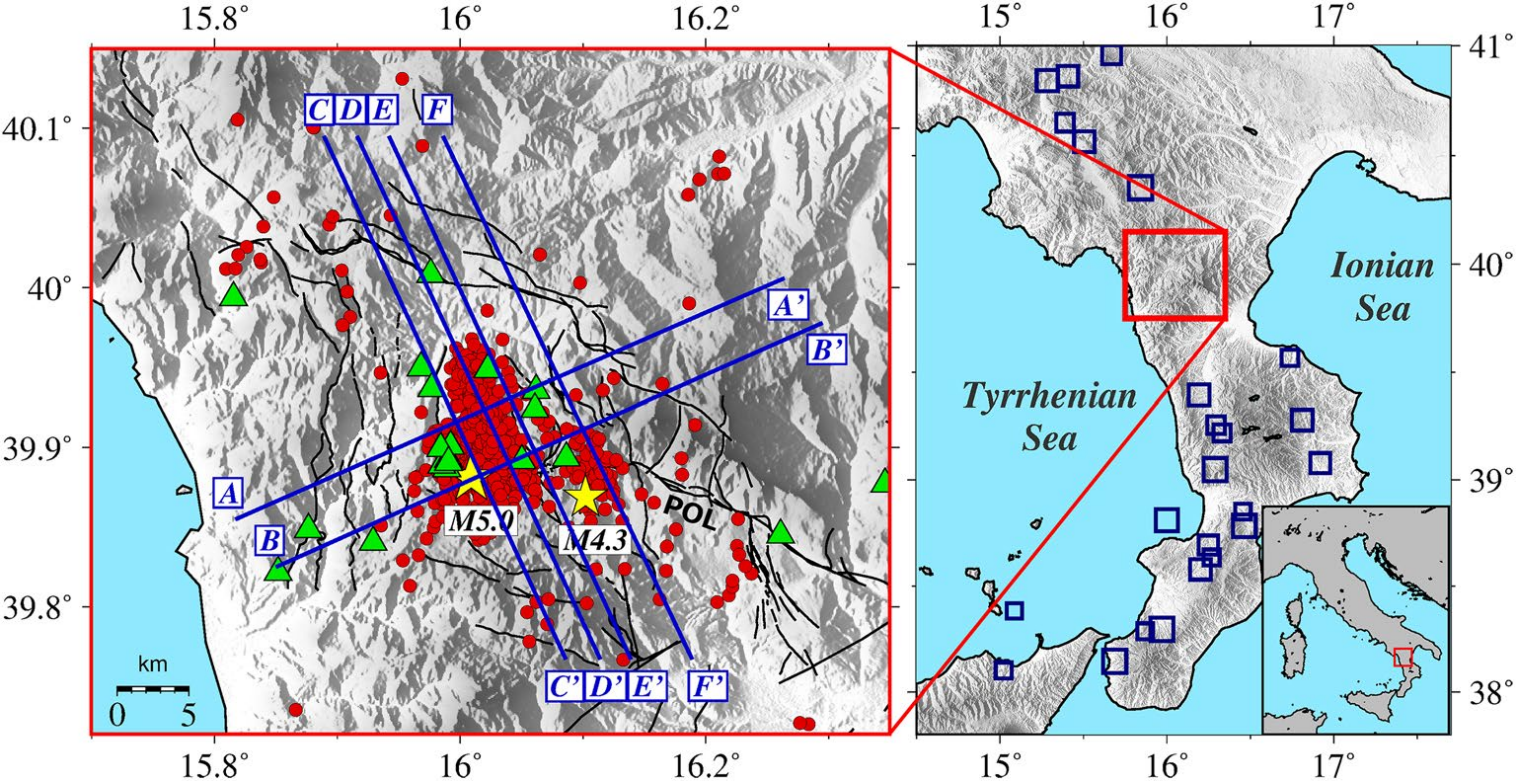
The left panel shows a zoom on the Pollino seismic gap region (right panel, red square) and aseismic region relative to historical earthquakes of M_L_ > 6 (right panel, blue squares, CPTI15^15^). Red circles show the earthquakes used in this work (located by Napolitano et al.^11^), while green triangles show permanent and temporary seismic station locations. The yellow stars show the locations of the two largest events of the sequence (M_L_ 5.0 and M_L_ 4.3), while the black lines show the fault traces (taken from Brozzetti et al.^8^). The blue segments represent the trace of the six vertical cross-sections through the final absorption and velocity, corresponding to and named after those shown by Cirillo et al.^10^.

Seismic gaps are areas where strong earthquakes are expected, but no significant historical and instrumental seismicity has been recorded^1^. Understanding the crustal characteristics of these gaps is essential for risk assessment and mitigation within the broader framework of seismic hazard. Despite intense seismic activity in southern Italy, the Mt. Pollino region (Fig. 1) shows sparse historical documentation of major earthquakes^2^, likely a consequence of its low population density^3^. On the contrary, paleo seismological studies have shown clear evidence that M 6.5–7 earthquakes occurred along two seismogenic structures, the Pollino and Castrovillari faults^3^. The contrasting evidence from paleo-, historical and present-day seismicity makes the Mt. Pollino area one of the most significant seismic gaps in Italy^4^.

Starting from October 2010, more than 10,000 small-to-moderate earthquakes struck the area across four years. The sequence developed as a combination of swarm-like (75%) and mainshock-aftershock (25%) events^5^. Seismicity spread across a wider eastern and a smaller western volume. The strongest earthquakes occurred years after the beginning of the sequence: a M_L_ = 4.3 on 28th May 2012 within the eastern volume, and the main event of the sequence, a M_L_ = 5.0 on 25th October 2012, within the western volume.

The Pollino region actively deforms at a rate slower than the Southern Apennines (2–2.5 mm/yr^6^). The inversion of geodetic time series identified a transient slip along the M_L_5.0 fault plane. The slip started 3–4 months before the earthquake, had the same focal mechanism, lasted one year, and released a magnitude equivalent to an M_W_5.5^7^. Geological models of the structures involved in the 2010–2014 sequence inferred a much higher seismogenic potential than the one released^8–10^, and the role of the Pollino fault as a barrier for the southward migration of the seismicity^10^.

The 2010–2014 Pollino sequence has provided geophysical data to image and model the corresponding seismic gap. 3D travel-time tomography^11,12^ has imaged the crustal structure of the seismogenic volume at high resolution. Clusters of repeaters characterized by similar waveforms have been found and relocated by Napolitano et al.^13^ along the M_L_ 5.0 fault plane. These events were caused by slow slip events similar to the transient deformation event detected by Cheloni et al.^7^, and favored by pore-pressure increases in fluid-saturated fault networks^14^. Shear wave splitting analysis^15^ highlighted strong crustal anisotropy and depicted a flower structure that played the role of preferential pathways for fluid migration, confirmed by recent geological models^10^. Direct-wave attenuation tomography recognized the volumes of two fluid reservoirs feeding the sequence and a low permeability volume topping the sequence above the M_L_ 5.0 fault plane^16^. Napolitano et al.^17^ mapped high scattering and absorption zones across the Pollino range at multiple frequencies, discriminating larger healed faults associated with high-magnitude earthquakes from fluid-filled connected fault systems linked with recent seismicity. Hundreds of focal mechanisms^13,18^ have been computed and used to perform Focal Mechanisms Tomography^19^, detecting an excess of pore pressure at the M_L_ 5.0 location and a possible mechanism of diffusion, which has played a key role in the development of the sequence. The extension of the volumes responsible for this overpressure and their relation to geophysically- and geologically-reconstructed structures is currently unknown.

Scattering and absorption measurements have proven reliable proxies for the spatial extension of faults, thrusts and fluid reservoirs across tectonic, volcanic and hydrothermal settings^17,20–24^. Scattering marks tectonic interactions and lithological contrasts^24^ due to mechanisms of wave trapping that increase energy across the earthquake coda^25,26^. While fluid content is a primary controller of seismic absorption^27^, rock physics and numerical studies have proven the sensitivity of this parameter to strain rate^25,28^ and pore space topology^29^.

Scattering and absorption tomography can use the entire envelope, the delay of its peak, or its coda decay as data^20,30–32^. They are becoming a standard combination in seismology, given their sensitivity to different structures and processes within the same volume. Across mountain chains, they could separate fault networks from sedimentary basins^31,33^, delineating tectonic interactions^34^ and detecting lithological contrasts and pathways for fluid migrations^23^. Reiss et al.^21^ revealed fractures and fault networks using peak delay, while absorption mapped sills, deformed heated pathways of dikes and the along-fault reservoir of fluids feeding a carbonatic volcano. The dependency of absorption on heat transfer and deformation makes absorption an efficient marker for magma and fluid storage and propagation at depth^21,35,36^, while scattering has proven its potential in detecting controllers of seismic sequences across fault networks^24^.

Scattering and absorption attributes have been recently studied and calibrated at smaller Earth scales and in the laboratory. Di Martino et al.^22^ demonstrated that peak delay contrasts mark faults and fluid pathways at 10 s of meters scales across the Solfatara crater, inside Campi Flegrei caldera. Through laboratory deformation experiments, King et al.^25,26^ fine-tuned the peak delay method to detect high-scattering fault segments and low-scattering pressurized zones, where aftershocks are most likely to occur. This evidence allowed^24^ to map and interpret the control of thrusts and pore pressure on the Amatrice-Visso-Norcia seismic sequence. Di Martino et al.^29^ finally demonstrated the control of pore-space topology on the entire waveform, and specifically peak delays and coda decays, a promising result considering the importance of excess pore pressure on the seismic sequence^19^.

In the present paper, we provide the first 3D scattering and absorption images of the Mt. Pollino seismic gap area (Fig. 1). After comparison with geology, we identify the low-scattering structures that have controlled the development of the sequence. In combination with previous velocity imaging, absorption tomography detects the highly-strained overpressurized volumes causing both deformation transients and seismic sequences. The results are discussed in the framework of the existing geophysical signals and the processes acting to generate them.

## Results

**Figure 2:**
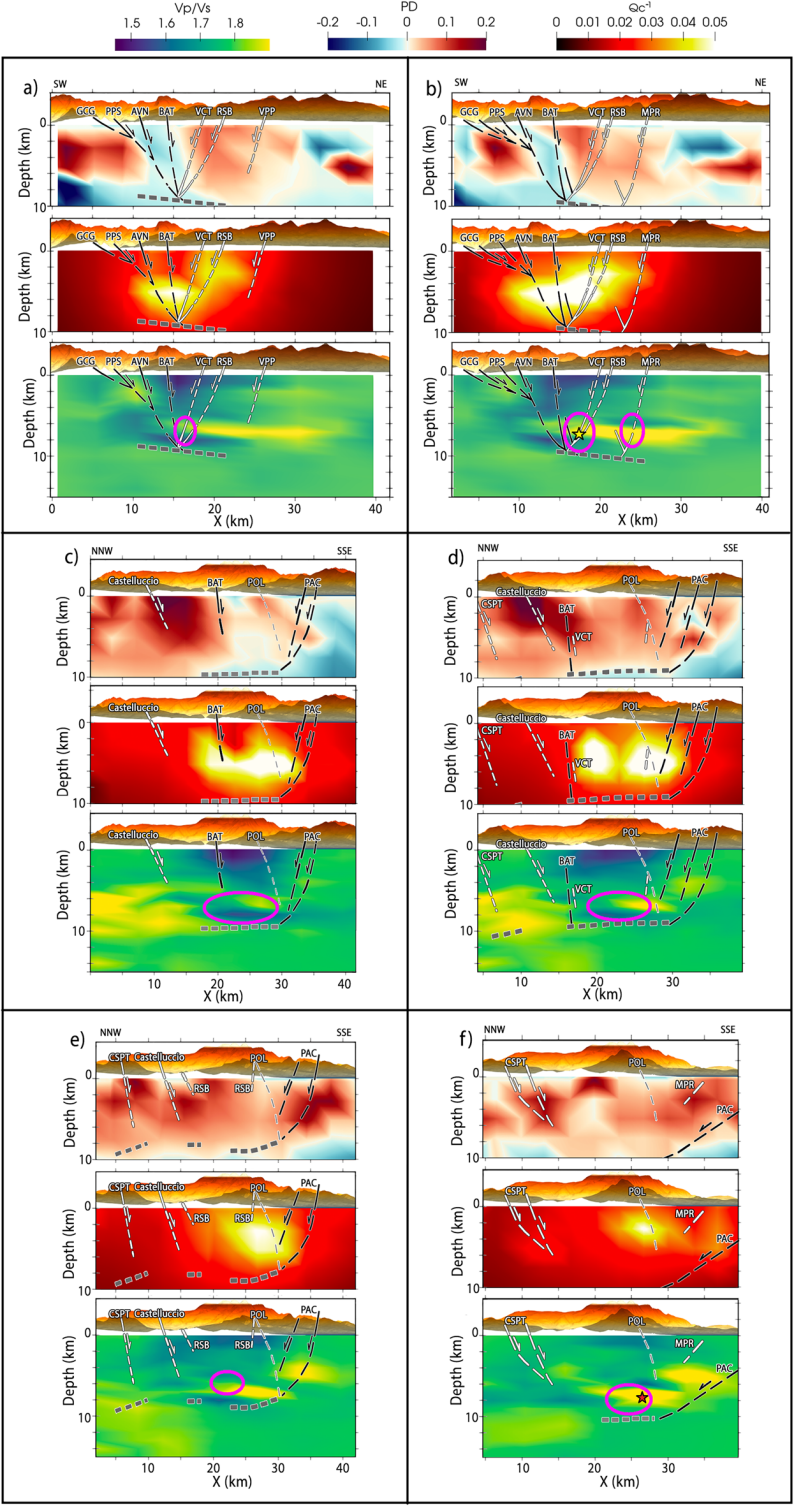
Vertical cross sections along SW-NE (panels **a** and **b**) and NNW-SSE directions (panels **c**–**f**) of scattering (peak delay—PD, top panel), absorption (Q_c_^−1^, middle panel), and Vp/Vs (bottom panel, from Napolitano et al.^11^). Inferred fault traces by Cirillo et al.^10^, with east- (west-)dipping faults colored in black (white): GCG: Gada–Ciagola fault; PPS: Papasidero fault; AVN: Avena fault; BAT: Battendiero fault; VCT: Fosso della Valle–Campotenese fault; RSB: Rotonda–Sambucoso; MPR: Morano Calabro–Piano di Ruggio fault; VPP: Viggianello–Piani del Pollino fault set; POL: Pollino fault; PAC: Monte Palanuda–Campolungo fault; Castelluccio: Castelluccio Fault; CSPT: Castello Seluci–Piana Perretti–Timpa della Manca fault. All profiles are cut above sea level, with shallower anomalies shown in Fig. 3. Magenta ellipses represent the Mt. Pollino sequence location in each slide, while the red and the yellow stars represent the ML 4.3 and the ML 5.0, respectively.

**Figure 3:**
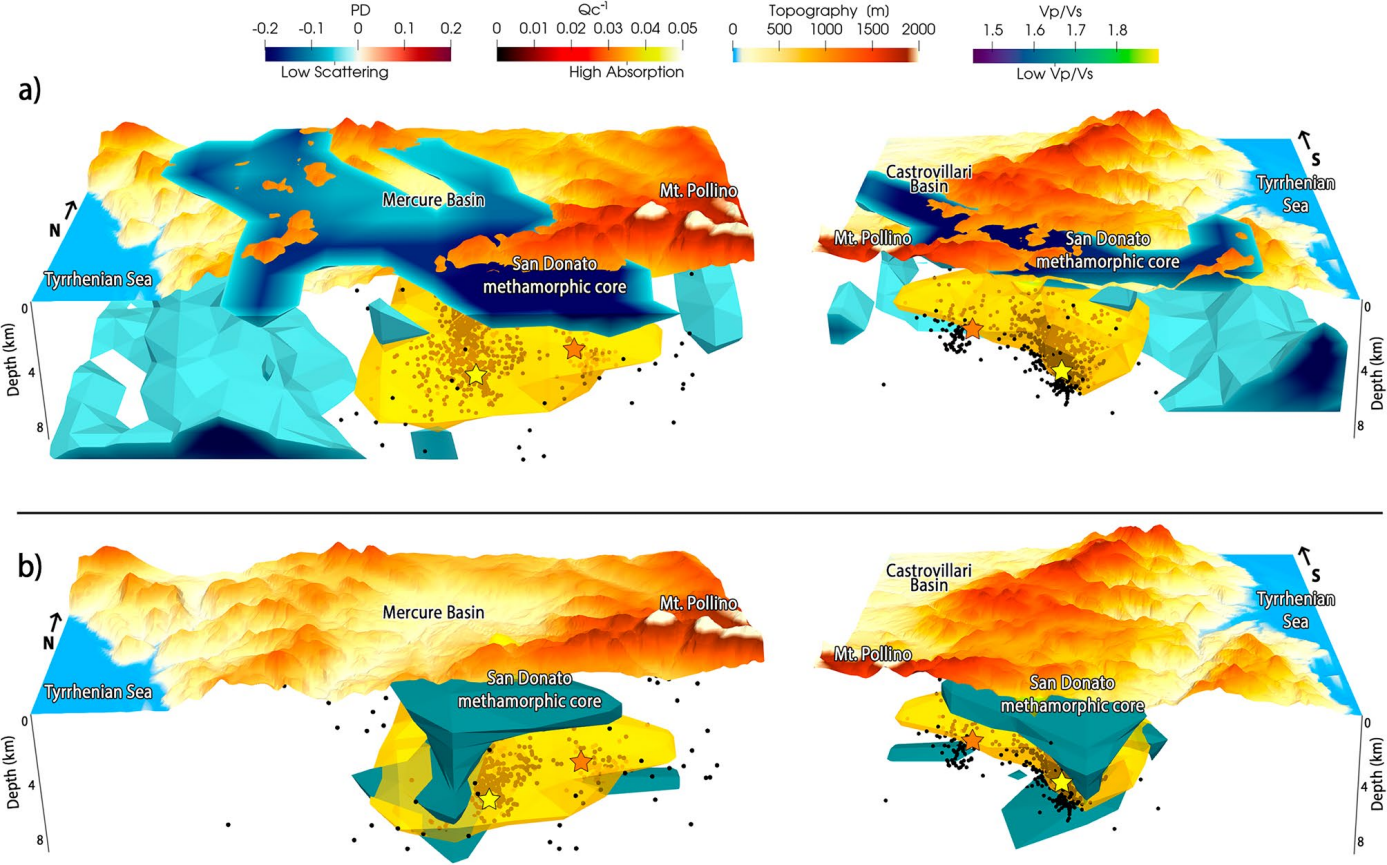
3D bird-eye views, from south to the north on the left hand side, and from north to the south on the right hand side, on the main anomalies discussed in the text. Specifically, we show the high absorption (Q_c_^−1^) anomaly compared with scattering (PD) in panel (**a**) and Vp/Vs in panel (**b**). The seismic events of the Mt. Pollino sequence used in this work are shown as black dots, while yellow and red stars represent the ML 5.0 and ML 4.3 event locations, respectively. In panel a) the high-absorption body (Q_c_^−1^, yellow isosurface) is topped by the low-scattering anomaly (PD, dark blue). Panel (**b**) shows the same anomaly topped by the low Vp/Vs anomaly (dark green) recognized as the San Donato metamorphic core. Surficial low-scattering anomalies (dark blue color in panel **a**), above sea level) extend to both the Mercure and Castrovillari sedimentary basins, while deep wide low-scattering anomalies (panel a) constrain the high-absorption anomaly to the west.

**Figure 4:**
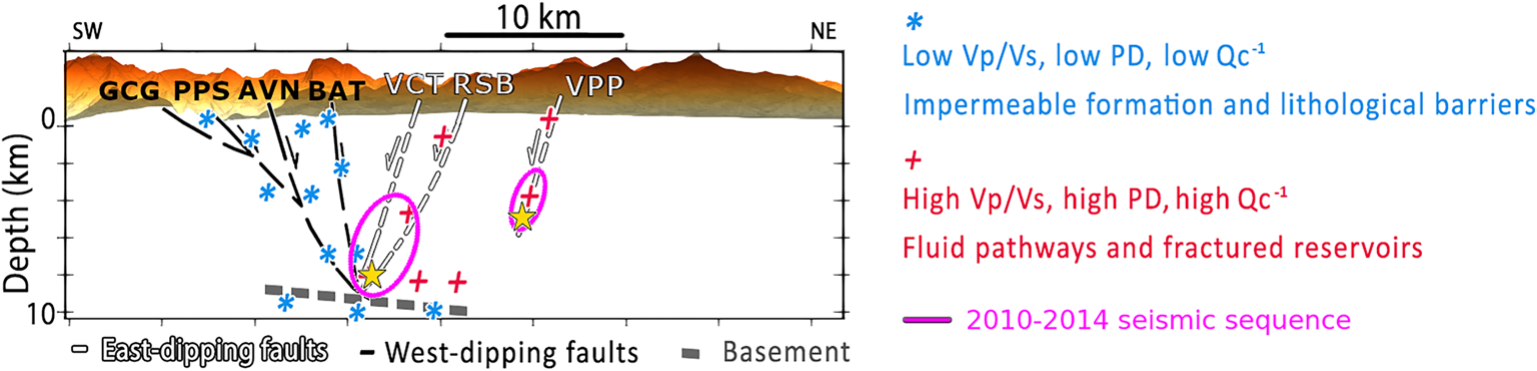
Schematic interpretation, as described in the text, of the main findings, replicating the AA' cross-section of Fig. 2. E-dipping faults and basement, characterized by low Vp/Vs, low peak delay and low Q_c_^−1^, that have been interpreted as impermeable formations and lithologic barriers for fluid migration are shown as light blue asterisks. W-dipping faults, interpreted as fluid pathways during the Mt. Pollino sequence, and as fluid reservoirs at 8–9 km depth, characterized by high V_P_/V_S_, high peak delay and high Q_c_^−1^, are marked with red crosses. Magenta ellipses represent the location of the 2010–2014 Pollino sequence, and the two stars are the ML5.0 event on the SW and the ML4.3 event on the NE. Abbreviations of the names of the faults are available in the caption of Fig. 2.

In Fig. 2a–f, we plot regionalized peak delay (PD) variations corrected from epicentral distances at 1.5 Hz (low scattering anomalies in blue—high scattering anomalies in red) in the top panels, absorption is shown as inverse coda attenuation (Q_c_^−1^) variations at 1.5 Hz in the middle panels, and the Vp/Vs model computed by Napolitano et al.^11^ is shown the bottom panels. We interpret scattering and absorption maps along the same cross-sections discussed by Cirillo et al.^10^, just overlapping their inferred faults on the maps resulting from our analysis. Their 3D fault models have been built by combining high-quality hypocenters of the events of the 2010–2014 seismic sequence, located through a double-difference relative location technique^37^, and structural-geological data of identified Quaternary faults in the Mt. Pollino region ^8,38^. East-dipping (black) and west-dipping (white) faults, the Pollino fault (POL in light grey), and the basal detachment (dark grey) are overlaid to each vertical cross-section (Vp/Vs, scattering and absorption) as inferred by Cirillo et al.^10^. The name abbreviations of the faults described in this work are in the caption of Fig. 2. Figure 3 provides 3D northward and southward views on the spatial relation between scattering and absorption (a) and Vp/Vs and absorption (b) models. Finally, Fig. 4 sums up the most significant findings in a schematic interpretation.

There are clear spatial relations between scattering anomalies and tectonic features. Figure 2a,b shows that the three SW-dipping structures (white dashed lines, VCT, RSB and MPR) involved in the sequence are marked by high scattering, while the NE-dipping structures (black dashed lines, GCG, PPS, AVN and BAT) are marked by low scattering features. Sharp scattering contrasts along the GCG and BAT NE-dipping faults constrain a low-scattering volume within two prevalently high-scattering anomalies, of which the one west of GCG/PPS is at the edge of the well-resolved zone, thus not interpreted. Figure 2c,d shows that the highest peak delays across the model characterize the volumes NNW of BAT, especially around the Castelluccio faults.

The peak delay model also depicts a horizontal layer which underlies the entire sequence, separating high scattering down to ~ 10 km depth from deeper low-scattering structures (Fig. 2c–e). The PAC fault network appears as a clear structural contrast in scattering, with its footwall imaged as low scattering anomalies bending to surface following the fault dips (Fig. 2c,d,f). Our results also highlight a scattering contrast, likely following a low-angle structure, immediately after PAC (Fig. 2d). These wide low-scattering anomalies at 4–8 km depth contour the western and SW part of the western seismic volume (Fig. 3a). Finally, a shallow low-scattering layer ~ 1–2 km thick extends from the NW Mercure basin, across the San Donato metamorphic core, to the SW Castrovillari basin (Fig. 3a).

High absorption anomalies concentrate within the 2010–2014 seismic volume (Fig. 2, middle panels), especially within and above the western seismic volume (Fig. 3a,b). Figure 2a,b shows the highest absorption values between 5 and 6 km depth, at the junction between the E-dipping BAT and W-dipping VCT faults. The anomaly spreads following the M_L_5.0 seismogenic volume at depth but reaches the surface exclusively across the W-dipping VCT and RSB faults. The anomaly appears confined to the north by the BAT fault and to the south by the POL/PAC structures (Fig. 2c,d). The only fault structure dipping consistently within the high-absorption body is the Pollino fault (POL—Fig. 2c–f). Low Vp/Vs anomalies sandwich the high-absorption volume and correspond to the shallow San Donato metamorphic core and the deep detached basement (Fig. 3b).

## Discussion

The sharp scattering contrasts along the GCG and BAT fault planes (Fig. 2a,b) delineate a low-scattering volume comprising most east-dipping faults (Fig. 2a,b). Their rheological differences relative to west-dipping faults^10^ act as a natural barrier to seismicity, resulting in a more compact medium if compared with the one affected by the Mt. Pollino seismic sequence. In contrast, all faults involved in the sequence (VCT, RSB, MPR) show high scattering, a by-product of intense strain and fracturing^25^ that occurred during the long and intense seismic sequence. High scattering values marked the swarm area at high frequencies in the previous 2D scattering and absorption maps^17^, highlighting the presence of highly fractured volumes. Further north-east, in the area at the edge of resolution at the surface, but well resolved at larger depths, we found a second scattering contrast east of VPP and MPR that seems to be likely related to a structure not mapped by recent geological models.

Figure 2c–e shows sections oriented NNW-SSE progressively moving NE. The primary scattering contrast matches the PAC fault system and continues horizontally at a 9–10 km depth, contouring the volume involved in the 2010–2014 sequence. The contrast matches the basal detachment inferred by Cirillo et al.^10^, i.e., the depth above which more than 90% of the events in the sequence occurred. This detachment primarily follows the PAC fault horizontally, but it deviates to a gentler dip further NE (Fig. 2d,e), which makes the accommodation of basal detachment more plausible. This structure, called Rossale fault (RSL), was mapped by Brozzetti et al.^8^ and shown in the plan by Cirillo et al.^10^ but was not reported in their cross sections.

Scattering anomalies reach the highest values north of the 2010–2014 seismic sequence, between the Castelluccio, CSPT and BAT faults, in relation with average-to-low Vp/Vs and low absorption (Fig. 2c–e). This highly-fractured region is the location of large magnitude historical events: a M_W_ 5.1 in 1894, a M_W_ 4.7 in 1980, a M_W_ 4.7 in 1988 (CPTI15^4^) and the M_W_ 5.6 Mercure earthquake sequence in 1998^38^. Around 200 earthquakes developed within the first 6 km depth during this sequence, leaving a high-scattering signature indicative of a highly-fractured volume. Similar patterns of high scattering and low absorption at 1.5 Hz and 3 Hz have also been observed by Napolitano et al.^17^ and related to fractured volumes. In this region of high total attenuation^16^ such high-scattering and low-absorption characteristics mark the inferred location of two carbonate platforms, the Apennines Platform overlapping at 5 km depth the deeper Inner Apulian Platform.

Although the seismicity used to obtain the peak delay maps reaches a maximum depth of 10 km, based on the seismic ray trajectories (Figs. S2 and S3) we observe a horizontal scattering contrast (Fig. 2c,d). However, interpreting the low scattering underlying the sequence is difficult because of the lack of, for example, deep wells for oil exploration, which has been forbidden in this National Park since the early 1990s. Nevertheless, the 9–10 km depth contrast matches the low Vp/Vs anomaly underlying the sequence^11^. The anomaly is 1 km shallower than the basal detachment inferred by Cirillo et al.^10^ because of the differences in earthquake localizations between the two papers but both likely represent the same lithological structure.

This change in lithology underlies the primary high-absorption volume, west of Mt. Pollino and mostly spreading within and above the western seismic volume (Fig. 3a,b). High absorption appears compressed between the detached basement and the shallow low-scattering, low Vp/Vs and low-absorption features (e.g., Figs. 2a and 3a,b) of the San Donato metamorphic core^8,39,40^. This core had been already identified as a low direct wave attenuation body and interpreted as impermeable formations^16^. The high-absorption volume corresponds to the highly-fractured carbonates extending between 4 and 8 km depth under the core. Its depth contrast marks fluids within the carbonates, confined by the impermeable behavior of the shallow metamorphic rocks.

The absorption confinement at depth is particularly evident across the western seismogenic volume (Fig. 3b) where most of the 2010–2014 seismicity occurred^5^. High attenuation values have been also found in the same seismogenic volume by means of total attenuation^16^. From SW to NE (Fig. 2a,b) high absorption focuses between W- and E-dipping faults, a consequence of the intense strain at the base of the faults^28^ and fluids that permeate the highly-fractured carbonates. While the high-absorption anomaly remains confined between 4 and 8 km depth along BAT, it rises toward the surface following VCT-RSB, and more generally the W-dipping faults (Fig. 4). The San Donato metamorphic core (1–3 km depth) apparently blocked fluid propagation toward the surface only across the E-dipping faults, forcing them to migrate toward the W-dipping VCT and RSB faults (Fig. 4). The very few seismic events located at the deepest edge (8–9 km depth) of the east-dipping structures along the AVN and BAT faults^8, 10^ confirm the interpretation of the low scattering volume, which characterizes these structures, as a barrier for fluid propagation and seismicity migration. When moving NE (Fig. 2c–e) the E-dipping BAT fault (to the north and west) and the PAC fault system (to the south) still appear to be natural barriers to deep fluid migration. The only recognised fault dipping within these high-absorption, saturated volumes remains the Pollino fault (POL, Fig. 2c–f).

These fluids are the most likely source for the excess pore fluid pressure evidenced by De Matteis et al.^19^ at similar depths. Their confinement could have favored the occurrence of the wide aseismic slip^7^, and caused clusters of repeaters characterized by similar waveforms along the M_L_ 5.0 fault plane^13^. While low Vp/Vs best identifies the metamorphic core (Fig. 3b) the shallow low-scattering features extend to the first kilometer of both the Mercure and Castrovillari basins. These basins appear as a fluid-saturated barrier north and southeast of the pressurized high-absorption volumes, a behavior previously observed in low-scattering shallow Triassic formations of fluid overpressure during the Amatrice-Visso-Norcia sequence^24^.

Fluid inputs within the high-absorption volumes will increase pore fluid pressure across fluid-filled cracks and around faults^5,19,41^, resulting in seismicity confined between depths of 4 and 8 km, and the main events located at the lowest boundary of the high-absorption saturated volume (Fig. 3b). This behavior suggests that poro-elasticity^7,19^ and lithological barriers^10,16^ have played a primary role in the release of stress and development of the sequence^5^. Even if stress and fluids had migrated east, this migration appears to have remained constrained by impermeable formations, with most of the seismicity clearly focusing at the bottom of the high-absorption volume (Figs. 3a,b and 4). Modeling the role of this pressurized volume on deformation signals, especially its ability to quantify the energy release associated with both seismic^5^ and aseismic^7^ stress, appears central to clarify if similar processes might act across the rest of the seismic gap.

## Conclusions

Our tomographic models identify and characterize the pressurized fluid-saturated volumes and lithological barriers causing a seismic sequence across one of the most prominent seismic gaps in Italy. Seismic attenuation mechanisms detect metamorphic, basement and fault structures sealing fluids within the mountain range and its fault networks and redistributing stress across the sequence. The characterization of different attenuation mechanisms provides a highly-resolved 3D picture of the sealed fluid-filled volumes responsible for most of the seismicity and long-lasting aseismic transients. Seismicity, especially mainshocks, appear to contour these volumes, giving a geophysical signature to mechanisms of excess pore-fluid pressure and stress transfer inferred by previous studies.

Scattering attenuation detects from high scattering fractured carbonates to basal detachment and impermeable shallow and deep layers constraining fluid migrations within metamorphic cores and saturated sedimentary basins. This is expected from the recent rock physics characterization of its attribute and applications across volcanic fields, mountain ranges and other fault networks supporting the strict connection between low scattering anomalies and locked or highly-pressurized zones. Seismic absorption instead shows potential to detect and characterize fluid-filled reservoirs and saturated media at a level of detail rarely obtained across fault networks. The joint analysis with field geology results remains central to a correct interpretation of the recovered anomalies. As scattering and absorption tomography are largely independent of the velocity structure, their comparison with highly-resolved velocity maps and seismic source studies offers a complementary geophysical approach to fault and reservoir characterization and a stepping stone to understand the wider-scale processes leading to regions of seismic gap.

## Methods

The total attenuation of seismic waves in a medium is related mainly to two mechanisms: seismic scattering and absorption^42^. Scattering and absorption contribution to the total attenuation can be separated and mapped in space using two methodologies, i.e. the peak delay time^43^ and the attenuation of coda waves^44^, respectively. These two techniques are implemented in the open access code MuRAT 3.0 (https://github.com/LucaDeSiena/MuRAT), which allows measuring and modeling 3D variations of total attenuation, scattering and absorption in space, differently, for example, from other techniques such as the Multi Lape-Time Windows Analysis^45^. Indeed, while MLTWA allows to compute average scattering and absorption values, based on different theoretical assumptions than peak delay and attenuation of coda waves, MuRAT3.0 allows to obtain and test spatial images of these quantities. The code has already been applied in volcanic^21,22^ and tectonic regions^17, 23,24,33^.

We collected 864 earthquakes in a 100 km × 120 km × 10 km seismogenic volume, already located in the new 3D model for the Pollino area by Napolitano et al.^11^. These events are characterized by 1.7 < M_L_ < 5.0, by source-receiver distances ranging from 1 to 81 km and depths smaller than 10 km (Fig. 1). The earthquakes have been recorded at 32 permanent and temporary seismic stations operated between 2010 and 2014 in the area by three institutions: Università della Calabria (UniCal), Istituto Nazionale di Geofisica e Vulcanologia (INGV), and Deutsches GeoForschungsZentrum of Potsdam (GFZ) (Fig. 1. See Napolitano et al.^11^ for further details on the seismic network). The dataset comprises 10,039 waveforms for each component (NS, EW, Z), for a total amount of 30,117 waveforms. The P- and S-wave onsets have been manually picked by Napolitano et al.^11^.

A large number of seismic rays is ideal for the attenuation analysis if these rays sample the area more or less uniformly; however, earthquakes within the Pollino seismic sequence are primarily located in the same area and far exceed the surrounding seismicity. We applied a declustering procedure to avoid over-sampling of the central area affected by the seismic sequence and thus equalize the distribution of radii throughout the analysis area as much as possible. We separated each inversion block by factor 5 in the x, y and z directions and only kept those event-station pairs with the lowest uncertainty value relative to their coda-decay measurement. This procedure, first applied by Reiss et al.^21^, drastically reduces oversampling while stabilizing the measurements most affected by changes in signal-to-noise ratio (minimum ratio equal to 3). The final number of used waveforms after declustering is 3861 waveforms for each component. We applied a forward and backward 1–2 Hz (fc 1.5 Hz) band-pass Butterworth filter (fourth order). The envelopes were computed using a Hilbert transform, and a sliding window with a duration eight times the inverse of the central frequency was used to smooth them. The choice of a small central frequency (fc = 1.5 Hz) to perform our analysis allows us to highlight and interpret the larger structures within the seismogenic volume and, at the same time, to keep a reasonable number of waveforms to perform the inversion when dealing with absorption.

### Peak delay time computation and mapping

A clear sign of the scattering contribution to wave attenuation is the delay between the S-wave onset and the highest amplitude reached by the envelope, named *peak delay time*, which naturally broadens with increasing source-receiver distances^31,43^. Peak delay times (*t*^*pd*^*(r)*, in seconds) increases linearly as a function of the logarithm of the hypocentral distance (*R*_*H*_, in km), through the following relationship:

1 $$log_{10} t^{pd} \left( f \right) = A\left( f \right) + B\left( f \right) \cdot log_{10} R_{H}$$

with *A(f)* and *B(f)* the regression fit coefficients^46^. The hypocentral distance is calculated using a 3D velocity model^11^, in order to propagate the rays in the 3D grid with a ray-bending approach [35, from ^47^). The 3D velocity model^11^ available for the Mt. Pollino area and obtained using the same seismic dataset of this work, allow us to improve ray tracing, increasing the stability of the peak delay method, as theoretically demonstrated by De Siena et al.^20^.

The amount of scattering accumulated along the ray path is identified by measuring the calculated peak delay time of the ith waveform to the theoretical peak delay at the corresponding hypocentral distance:

2 $$\Delta log_{10} t\left( f \right) = log_{10} t_{i}^{pd} \left( f \right) - log_{10} t^{pd} \left( f \right)$$

Positive scattering variations ($\Delta log_{10} t\left( f \right)$ > 0) correspond to high-scattering zones interpreted as high heterogeneous crustal regions. Negative values, on the other hand, identify low-scattering zones, which are thought to be solid and compact zones. Fig. S1 in Supplementary material online shows the log–log plot of peak delay, within one standard deviation from the linear fit, as a function of the P-wave travel time for the Pollino area at $f_{c}$ = 1.5 Hz. To compute peak delays, the use of P- instead of S-wave onset after correction for the 3D Vp/Vs structure, has been theoretically applied and tested in volcanic setting by De Siena et al.^20^ (see their Fig. 4), and in a tectonic environment by Napolitano et al.^17^.

For each source-receiver ray we computed the peak delay time as the lag between the P-wave onset and the maximum S-wave amplitude^43^. Then we mapped the logarithmic variation of the P-wave peak delay time, assuming source-receiver sensitivity on rays, and used a conventional weighted regionalization technique^24,31,44^ on a grid with 2.5 km horizontally and 1 km vertically spaced nodes. The assumption of sensitivity on rays is valid, as always, only for infinite frequencies. Scattering defines a zone around the ray that gets wider as the frequency is lower. In each case, the presence of fractured/shear zones produces waves that go beyond this assumption, producing peaks that, at stations one wavelength from the fractured zone, dramatically increase the peak delay. These results have been proven at the rock scale by King et al.^25^ with experiments, and King et al.^26^ with modeling. For this reason, we use regionalization, a weighted average of the peak delay values of all those rays crossing a grid cell, and interpret it as positive and negative variations from the linear regression fit. The peak delay time stability has been assessed by computing the hit count map (Fig. S2), interpreting all those peak delay variations within blocks crossed by at least 20 seismic rays. For sake of completeness, we also show the ray coverage (Fig. S3) at different significant depths showing a good recovery of the 2010–2014 Pollino sequence area at each depth, and the peak delay maps at 1.5 and 3 Hz in Fig. S4 of the Supplementary material. The comparison shows that most of the peak delay anomalies, located within well-resolved area by the hit count (Fig. S2) are retrieved in both maps, unless expected changes in absolute value.

Recent laboratory and seismic modeling works have demonstrated that trapped waves will increase peak delay in correspondence with fractured fault zones^25,26^. These works prove that, at these scales, trapped and resonant waves drastically increase peak delays at a boundary, making it a marker of strain increase, fracturing (high peak delays) and overpressure (low peak delays). Despite the differences in scale, the ability of peak delays to mark "scattering boundaries", i.e., shear zones, barriers, and geological units, has been proven through the last years ^17,20,21,24,31^.

### Coda attenuation computation and mapping

Strong lateral differences in lithospheric characteristics may be imaged using coda wave attenuation, and a number of methods have been developed to evaluate these variations. Computing the energy density envelopes of coda waves is the first step that all of these algorithms share in order to get the coda quality factor, abbreviated Q_C_, for each waveform. The following equation^44^ represents the energy within the coda window, E(t,f), at fixed lapse-time t, in seconds from the origin time^48^, and frequency f:

3 $$E\left( {t,f} \right) = S\left( f \right) t^{ - \alpha } exp\left( { - 2\pi ftQ_{c}^{ - 1} \left( f \right)} \right)$$

where S(f) is the source term and α is a constant related to geometrical spreading, fixed to 3/2 in a multiple scattering interpretation. In this interpretation, Q_c_^−1^ ≈ Q_i_^−1^ if, after a few mean free times (mean time between two scattering events), the diffusion regime is reached^49^. The code MuRAT3D allows to solve the equation by using either a linearized or a grid search approach to measure $Q_{c}^{ - 1}$. We applied the linearization approach to solve Eq. (3). This approach, which has been proven to be computationally faster than grid search^17^ and equivalent to the linearised approach for signal to noise ratio (SNR) higher than 3 and coda window length larger than 10 s^50^ allows to measure $Q_{c}^{ - 1}$ for each recorded waveform using a straight-line fitting:

4 $$\frac{{ln\left[ {E\left( {t,f} \right) \cdot t^{\alpha } } \right]}}{2\pi f} = \frac{{ln\left[ {S\left( f \right)} \right]}}{2\pi f} - Q_{c}^{ - 1} t$$

We set the lapse time at 30 s for the whole dataset, which is a reasonable time for the coda window to reach the diffusive regime in the Pollino area, since Napolitano et al.^17^ showed that this regime is reached a few seconds after the S-wave arrival times in this area. The length of the window for the coda analysis is set at 15 s. Fig. S5 in the Supplementary material online shows the $Q_{c}^{ - 1}$ measurements obtained for each waveform (shown as blue circles in the upper panel) as a function of the ray length within a central frequency of 1.5 Hz. In the lower panel of the same figure we also provided a moving average and the standard deviation of $Q_{c}^{ - 1}$, computed on 500 points windows. To further confirm the stability of $Q_{c}^{ - 1}$ at different hypocentral distances, we first provided the histograms of $Q_{c}^{ - 1}$ (Fig. S6), assessing the normality of the distribution. Then we computed the average and standard deviation at each distance range (Table S1). The values are comparable within the measurement errors. We are thus confident that the $Q_{c}^{ - 1}$ measurements in the selected coda window are constant and independent within the selected ray length range^49^. It assumes average values within the selected ray length range of 0.0147 at 1.5 Hz.

Seismic absorption images in space have been obtained by applying an inversion scheme proposed by De Siena et al.^35^, that makes use of 3D multiple-scattering sensitivity kernels^51,52^ based on Paasschen’s equations^53^, independent of the choice of the 3D velocity model. This is because the quantities ruling the absorption sensitivity kernels are albedo (the dimensionless ratio of the scattering loss to total attenuation), and extinction length (the distance, in km, over which the primary S-wave energy is decreased by e−1), which are set to average quantities for similar tectonic regions. The sensitivity kernels require constant S-wave velocities in the computation. Consequently we have assumed Vs = 3.0 km/s, obtained from the average of the specific 3D Mt. Pollino velocity model^11^.We set the same grid defined for peak delay, with 2.5 km horizontally and 1 km vertically spaced nodes. The well resolved areas of the Q_c_^-1^ spatial variation maps have been assessed through checkerboard tests, shown at 2 and 5 km depths (Fig. S7), showing that the swarm area is well resolved. In addition, we also provided a spike test for the high absorption anomaly located in the correspondence of the M_L_ 5.0 seismogenic volume, at 5 km depth (Fig. S8). Both the resolution tests allow trusting the absorption results. As for peak delay, we also show for sake of completeness the Q_c_^-1^ maps at 1.5 and 3 Hz in Fig. S9 in the Supplementary Material online. The comparison shows that in the well-resolved areas (shown through the checkerboard and the spike tests in Figs. S7 and S8) the absorption anomalies are recovered, with an expected decrease in the Q_c_^-1^ absolute value for increasing frequency. Differences outside the well-resolved areas are not significant for the purposes of our analysis as they are not interpreted.

More uncertainties relate to Q_c_^-1^ as a marker of absorption. Starting from Calvet et al.^31^, there has been an intense effort of the community to define the "best" scattering and absorption solution useful for imaging with coda waves in a multiple-scattering leaking regime using radiative transfer theory and ray approximations^54–56^. This is an effect that wave-equation modeling has recently acknowledged by simulating wavefield across an oceanic basin. Nardoni et al.^57^ show the amount of energy lost through leakage into the mantle in this case but recognise the stability of Q_c_^-1^ as an absorption marker when the Moho contrast is deep enough, like at Pollino. On the other end of the spectrum, Di Martino et al.^22,29^ perform a calibration similar to King et al.^25,26^ for peak delay but also investigate Q_c_^−1^ with experiments and modeling. They demonstrate that the heterogeneity of an undeformed volcanic sample is sufficient to change the intermediate coda drastically. Still, its late-time coda decay remains a function of the fluid (in this case, air, in its pore space). This calibration is only partial: for definitive proof, one should conduct experiments with fluids of different saturation.

### Supplementary Information

Supplementary Information.

## Data Availability

Seismic waveforms used to perform the analysis are available in the Zenodo repository, https://doi.org/10.5281/zenodo.7982070, last access 30/05/2023.

## Code availability

The workspace used to perform analyses with the open-access MuRAT3D MATLAB© code is available at https://github.com/LucaDeSiena/MuRAT (MuRAT3.0, 2021).

## References

[1] Omori F (1909). Preliminary report on the Messina-Reggio Calabria earthquake of december 28, 1908. Bull. Earthq. Invest. Commun..

[2] Scionti V, Galli P, Chiodo G (2006). The Calabrian seismicity during the Viceroyalty of Naples: Sources silence or silent sources? The case of the strong 1744 earthquake. Boll. Geofis. Teor. Appl..

[3] Cinti FR, Cucci L, Pantosti D, D'Addezio G, Meghraoui M (1997). A major seismogenic fault in a ‘silent area’: The Castrovillari fault (southern Apennines, Italy). Geophys. J. Int..

[4] Rovida A, Locati M, Camassi R, Lolli B, Gasperini P (2020). The Italian earthquake catalogue CPTI15. Bull. Earthq. Eng..

[5] Passarelli L (2015). Aseismic transient driving the swarm-like seismic sequence in the Pollino range, Southern Italy. Geophys. J. Int..

[6] D’Agostino N (2011). Forearc extension and slow rollback of the Calabrian Arc from GPS measurements. Geophys. Res. Lett..

[7] Cheloni D (2017). Aseismic transient during the 2010–2014 seismic swarm: Evidence for longer recurrence of M ≥ 65 earthquakes in the Pollino gap (Southern Italy)?. Sci. Rep..

[8] Brozzetti F (2017). Newly identified active faults in the Pollino seismic gap, southern Italy, and their seismotectonic significance. J. Struct. Geol..

[9] Ferranti L, Milano G, Pierro M (2017). Insights on the seismotectonics of the western part of northern Calabria (Southern Italy) by integrated geological and geophysical data: coexistence of shallow extensional and deep strike-slip kinematics. Tectonophysics.

[10] Cirillo D (2022). Structural complexities and tectonic barriers controlling recent seismic activity in the Pollino area (Calabria–Lucania, southern Italy)–constraints from stress inversion and 3D fault model building. Solid Earth.

[11] Napolitano F (2021). Crustal structure of the seismogenic volume of the 2010–2014 Pollino (Italy) seismic sequence from 3D P- and S-wave tomographic images. Front. Earth Sci..

[12] De Gori P, Lucente FP, Govoni A, Margheriti L, Chiarabba C (2022). Seismic swarms in the Pollino seismic gap: Positive fault inversion within a popup structure. Front. Earth Sci..

[13] Napolitano F, Galluzzo D, Gervasi A, Scarpa R, La Rocca M (2021). Fault Imaging at Mt Pollino (Italy) from relative location of microearthquakes. Geophys. J. Int..

[14] Passarelli L, Selvadurai PA, Rivalta E, Jónsson S (2021). The source scaling and seismic productivity of slow slip transients. Sci. Adv..

[15] Pastori M (2021). The 2011–2014 Pollino seismic swarm: complex fault systems imaged by 1D refined location and shear wave splitting analysis at the apennines-Calabrian arc boundary. Front. Earth Sci..

[16] Sketsiou P, De Siena L, Gabrielli S, Napolitano F (2021). 3-D attenuation image of fluid storage and tectonic interactions across the Pollino fault network. Geophys. J. Int..

[17] Napolitano F (2020). Scattering and absorption imaging of a highly fractured fluid-filled seismogenetic volume in a region of slow deformation. Geosci. Front..

[18] Totaro C (2015). An intense earthquake swarm in the southernmost Apennines: Fault architecture from high-resolution hypocenters and focal mechanisms. Bull. Seismol. Soc. Am..

[19] De Matteis R (2021). Pore fluid pressure imaging of the Mt. Pollino region (southern Italy) from earthquake focal mechanisms. Geophys. Res. Lett..

[20] De Siena L, Calvet M, Watson KJ, Jonkers ART, Thomas C (2016). Seismic scattering and absorption mapping of debris flows, feeding paths, and tectonic units at Mount St. Helens volcano. Earth Planet. Sci. Lett..

[21] Reiss MC, De Siena L, Muirhead JD (2022). The interconnected magmatic plumbing system of the Natron Rift. Geophys. Res. Lett..

[22] Di Martino MDP, De Siena L, Serlenga V, De Landro G (2022). Reconstructing hydrothermal fluid pathways and storage at the Solfatara Crater (Campi Flegrei, Italy) using seismic scattering and absorption. Front. Earth Sci..

[23] Gabrielli S (2022). Fast changes in seismic attenuation of the upper crust due to fracturing and fluid migration: The 2016–2017 central italy seismic sequence. Front. Earth Sci..

[24] Gabrielli S (2023). Scattering attenuation images of the control of thrusts and fluid overpressure on the 2016–2017 Central Italy seismic sequence. Geophys. Res. Lett..

[25] King T, De Siena L, Benson P, Vinciguerra S (2023). Mapping faults in the laboratory with seismic scattering 1: The laboratory perspective. Geophys. J. Int..

[26] King T (2023). Mapping faults in the laboratory with seismic scattering 2: The modelling perspective. Geophys. J. Int..

[27] Barton N (2006). Rock Quality, Seismic Velocity, Attenuation and Anisotropy.

[28] Natale Castillo MA, Tesauro M, Cacace M (2022). How does seismic attenuation correlate to rheology of crustal rocks? Results from a numerical approach. Glob. Planet. Change.

[29] Di Martino MDP, De Siena L, Tisato N (2022). Pore space topology controls ultrasonic waveforms in dry volcanic rocks. Geophys. Res. Lett..

[30] Prudencio J, Del Pezzo E, García-Yeguas A, Ibáñez JM (2013). Spatial distribution of intrinsic and scattering seismic attenuation in active volcanic islands–I: Model and the case of Tenerife Island. Geophys. J. Int..

[31] Calvet M, Sylvander M, Margerin L, Villaseñor A (2013). Spatial variations of seismic attenuation and heterogeneity in the Pyrenees: Coda Q and peak delay time analysis. Tectonophysics.

[32] Del Pezzo E, Ibanez J, Prudencio J, Bianco F, De Siena L (2016). Absorption and scattering 2-D volcano images from numerically calculated space-weighting functions. Geophys. J. Int..

[33] Borleanu F, De Siena L, Thomas C, Popa M, Radulian M (2017). Seismic scattering and absorption mapping from intermediate-depth earthquakes reveals complex tectonic interactions acting in the Vrancea region and surroundings (Romania). Tectonophysics.

[34] Borleanu F, Petrescu L, Seghedi I, Thomas C, De Siena L (2023). The seismic attenuation signature of collisional orogens and sedimentary basins within the Carpathian Orogen. Glob. Planet. Change.

[35] De Siena L (2017). Source and dynamics of a volcanic caldera unrest: Campi Flegrei, 1983–84. Sci. Rep..

[36] Guardo R, De Siena L, Prudencio J, Ventura G (2022). Imaging the absorbing feeding and eruptive pathways of Deception Island, Antarctica. Geophys. Res. Lett..

[37] Waldhauser F, Ellsworth WL (2001). A double-difference earthquake location algorithm: Method and application to the northern Hayward fault, California. Bull. Seismol. Soc. Am..

[38] Brozzetti F, Lavecchia G, Mancini G, Milana G, Cardinali M (2009). Analysis of the 9 September 1998 Mw 5.6 Mercure earthquake sequence (Southern Apennines Italy): A multidisciplinary approach. Tectonophysics.

[39] Amodio Morelli M (1976). L’arco calabro-peloritam nell’orogene Appenninico-Maghrebide. Mem. Soc. Geol. Ital..

[40] Iannace A (2005). Structural setting and tectonic evolution of the Apennine Units of northern Calabria. Comptes Rendus Geosci..

[41] Amoroso O, Ascione A, Mazzoli S, Virieux J, Zollo A (2014). Seismic imaging of a fluid storage in the actively extending Apennine mountain belt, southern Italy. Geophys. Res. Lett..

[42] Sato H, Fehler MC, Maeda T (2012). Seismic Wave Propagation and Scattering in the Heterogeneous Earth.

[43] Takahashi T, Sato H, Nishimura T, Obara K (2007). Strong inhomogeneity beneath Quaternary volcanoes revealed from the peak delay analysis of S-wave seismograms of microearthquakes in northeastern Japan. Geophys. J. Int..

[44] Aki K, Chouet B (1975). Origin of coda waves: Surce, attenuation, and scattering effects. J. Geophys. Res..

[45] Fehler M, Hoshiba M, Sato H, Obara K (1992). Separation of scattering and intrinsic attenuation for the Kanto-Tokai region, Japan, using measurements of S-wave energy versus hypocentral distance. Geophys. J. Int..

[46] Gusev AA, Abubakirov IR (1999). Vertical profile of effective turbidity reconstructed from broadening of incoherent body-wave pulses—I. General approach and the inversion procedure. Geophys. J. Int..

[47] Block, L. V. (1991), Joint hypocenter‐velocity inversion of local earth- quakes arrival time data in two geothermal regions, Ph.D. thesis, Mass. Inst. of Technol., Cambridge.

[48] Havskov J (2016). Coda Q in different tectonic areas, influence of processing parameters. Bull. Seismol. Soc. Am..

[49] Calvet M, Margerin L (2013). Lapse-time dependence of coda Q: Anisotropic multiple-scattering models and application to the Pyrenees. Bull. Seismol. Soc. Am..

[50] Sketsiou P, Napolitano F, Zenonos A, De Siena L (2020). New insights into seismic absorption imaging. Phys. Earth Planet. Inter..

[51] Del Pezzo E (2018). Numerically calculated 3d space-weighting functions to image crustal volcanic structures using diffuse coda waves. Geosciences.

[52] Del Pezzo E, Ibáñez JM (2020). Seismic coda-waves imaging based on sensitivity kernels calculated using an heuristic approach. Geosciences.

[53] Paasschens JCJ (1997). Solution of the time-dependent Boltzmann equation. Phys. Rev. E.

[54] Wegler U (2004). Diffusion of seismic waves in a thick layer: Theory and application to Vesuvius volcano. J. Geophys. Res. Solid Earth.

[55] Margerin L (2017). Computation of Green’s function of 3-D radiative transport equations for non-isotropic scattering of P and unpolarized S waves. Pure Appl. Geophys..

[56] van Dinther C, Margerin L, Campillo M (2021). Implications of laterally varying scattering properties for subsurface monitoring with coda wave sensitivity kernels: Application to volcanic and fault zone setting. J. Geophys. Res. Solid Earth.

[57] Nardoni C, De Siena L, Cammarano F, Magrini F, Mattei E (2021). Modelling regional-scale attenuation across Italy and the Tyrrhenian Sea. Phys. Earth Planet. Inter..

