# Supplementary material for: Imaging overpressurised fracture networks and geological barriers hindering fluid migrations across a slow-deformation seismic gap
Source: Sci Rep. 2023 Nov 11;13:19680. doi: 10.1038/s41598-023-47104-w (PMC10640567; doi:10.1038/s41598-023-47104-w)
Supplement: Supplementary file 1 — Supplementary Information. [file 41598_2023_47104_MOESM1_ESM.docx]

**Supplementary material**

**
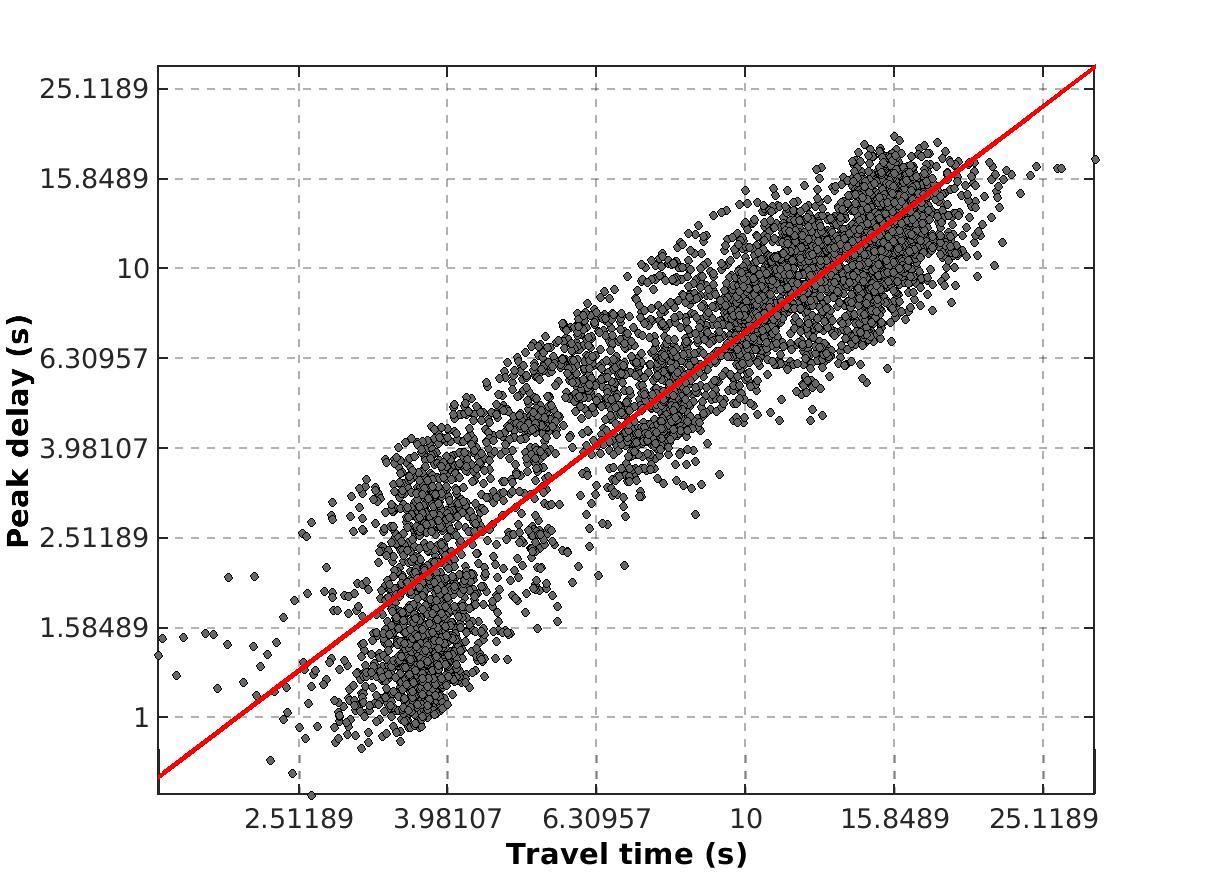
**

Figure S1. Log-log plot of peak delay as a function of the P wave travel time at 1.5 Hz. The regression fit coefficients are: A=-0.39, B=1.25.


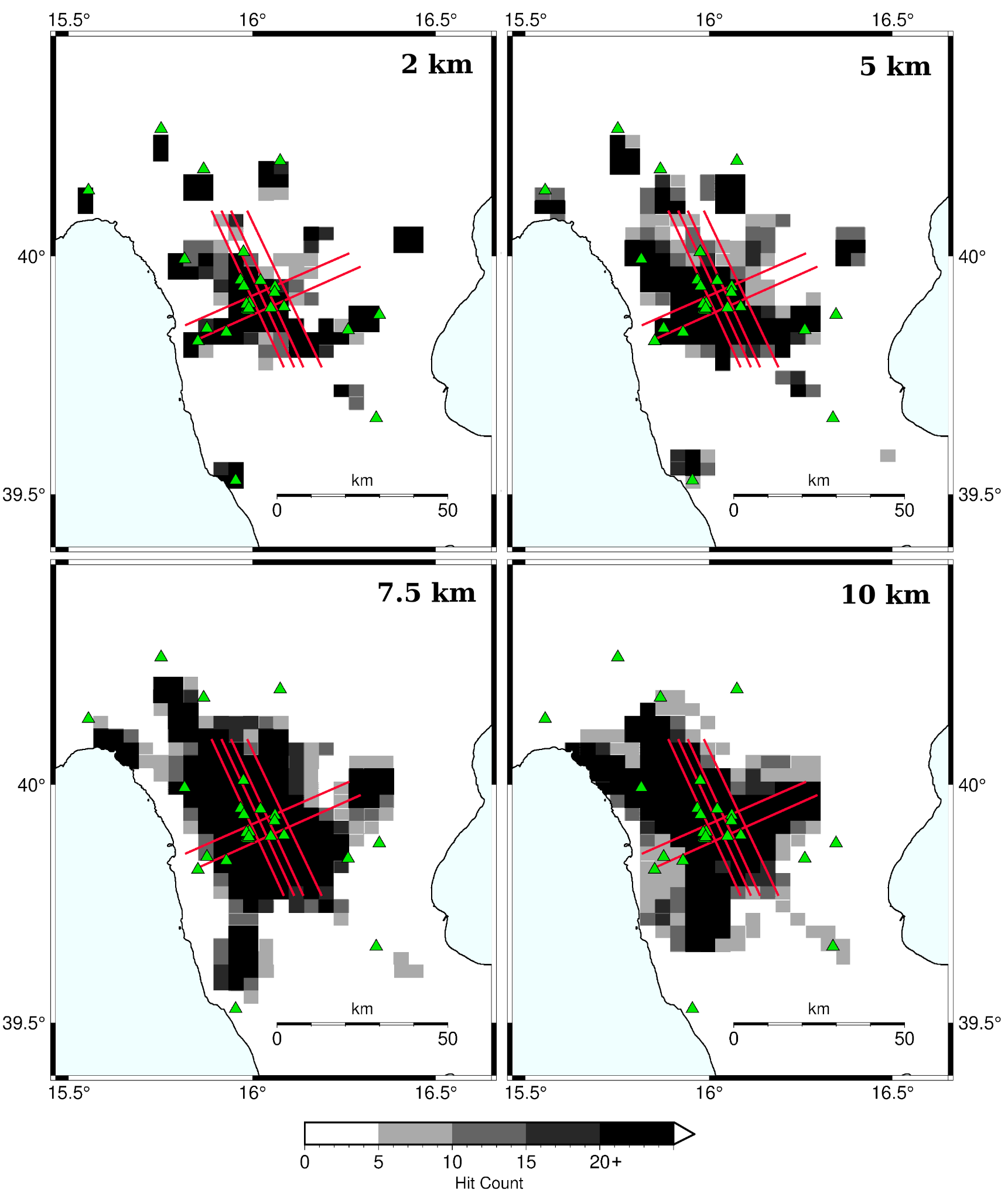


Figure S2. Hit count at different depths (2, 5, 7.5 and 10 km) showing the well resolved areas for peak delay maps. Blocks crossed by at least 20 rays are shown in black color, while we show blocks crossed by a smaller number of rays with lighter shades of gray (lighter for decreasing number of rays), leaving areas not crossed by any rays blank. Red lines represent the cross section locations shown in Figure 2.

Figure S3. Ray coverage of the Pollino area (black lines). Blue circles are the hypocenter locations and pink triangles are the seismic stations used in this work.
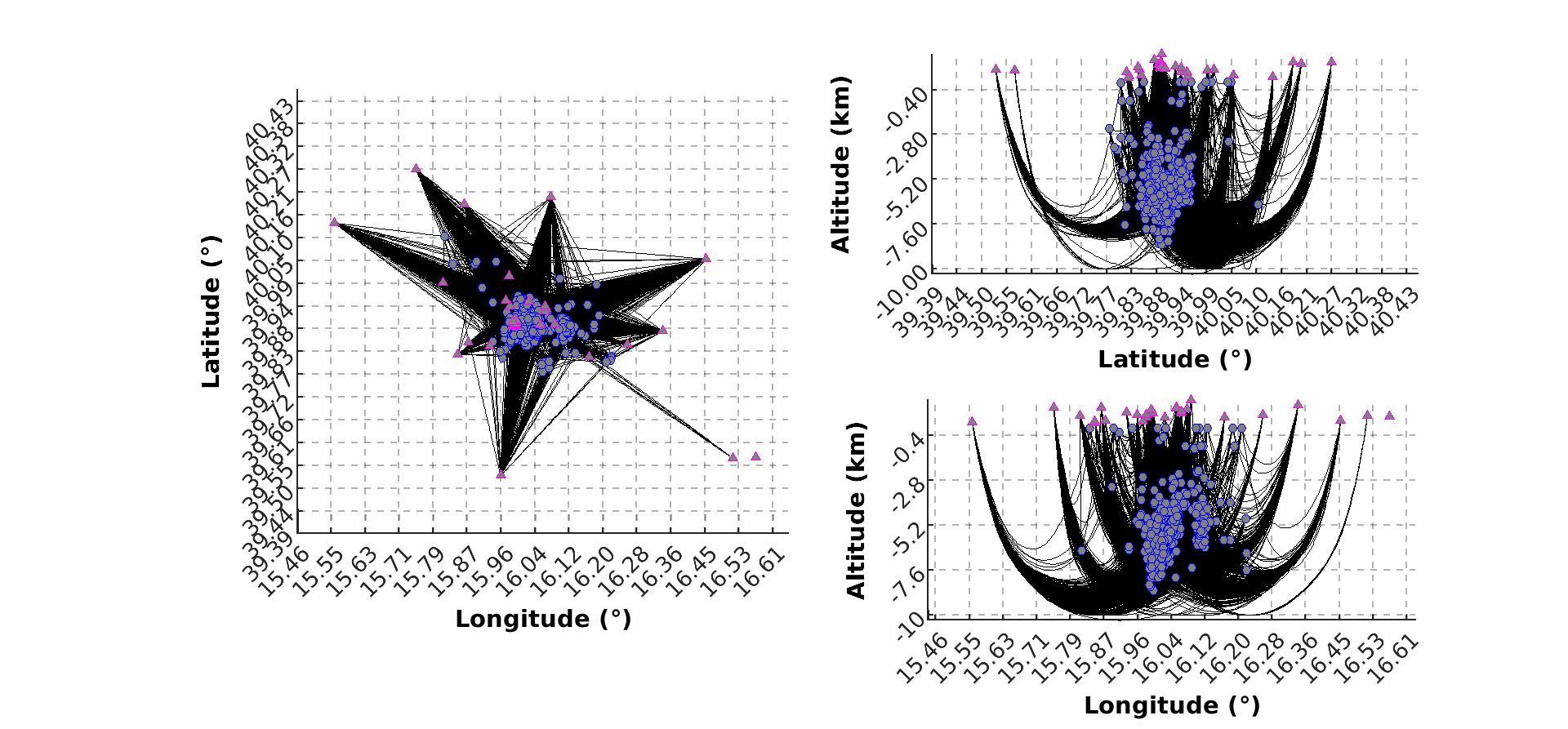


Fig. S4. Peak delay maps at different depths (2km, 5 km, 7.5 km and 10 km) computed using two central frequencies: 1.5 Hz, discussed in the main text, and 3 Hz, shown for comparison.
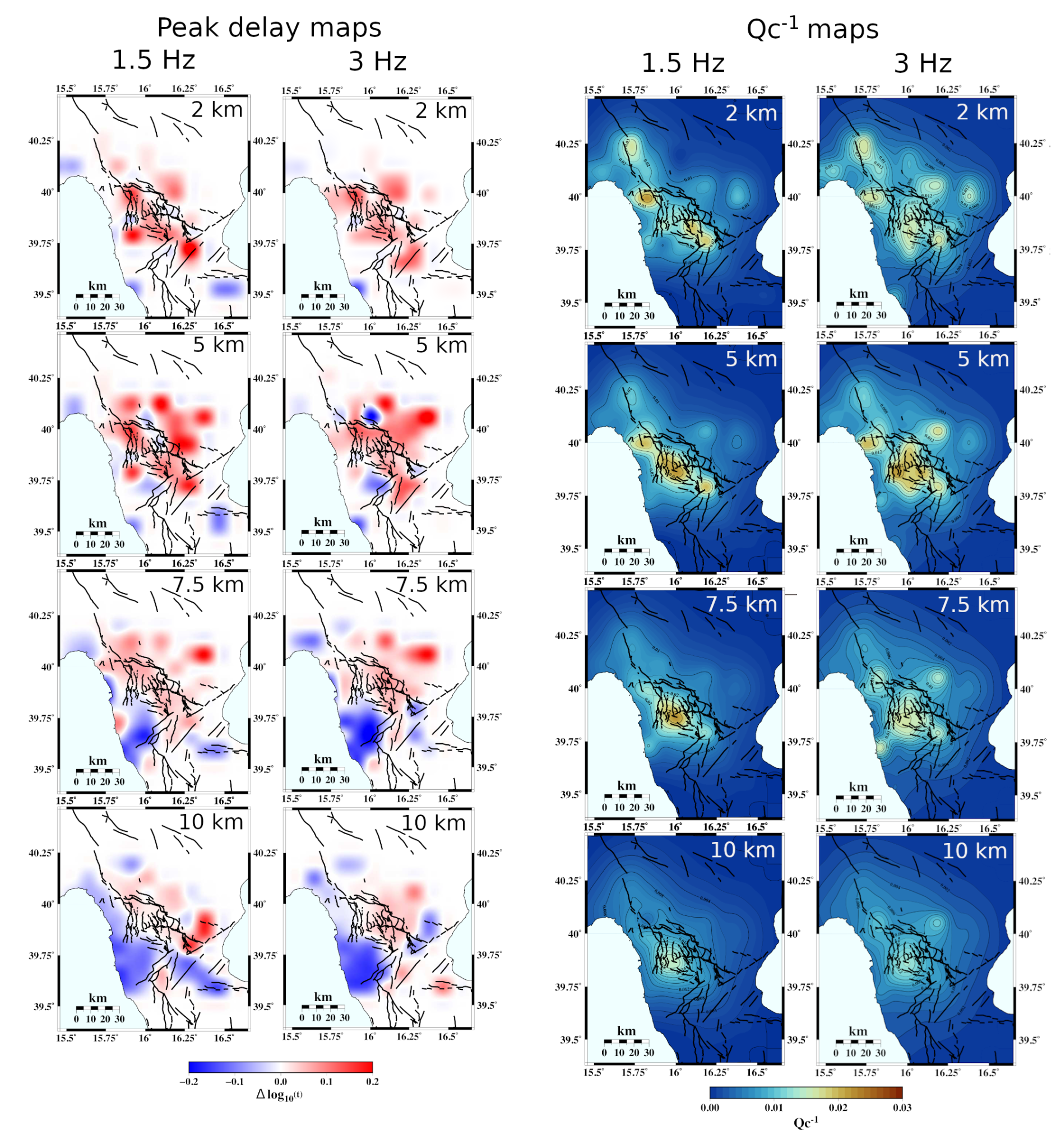


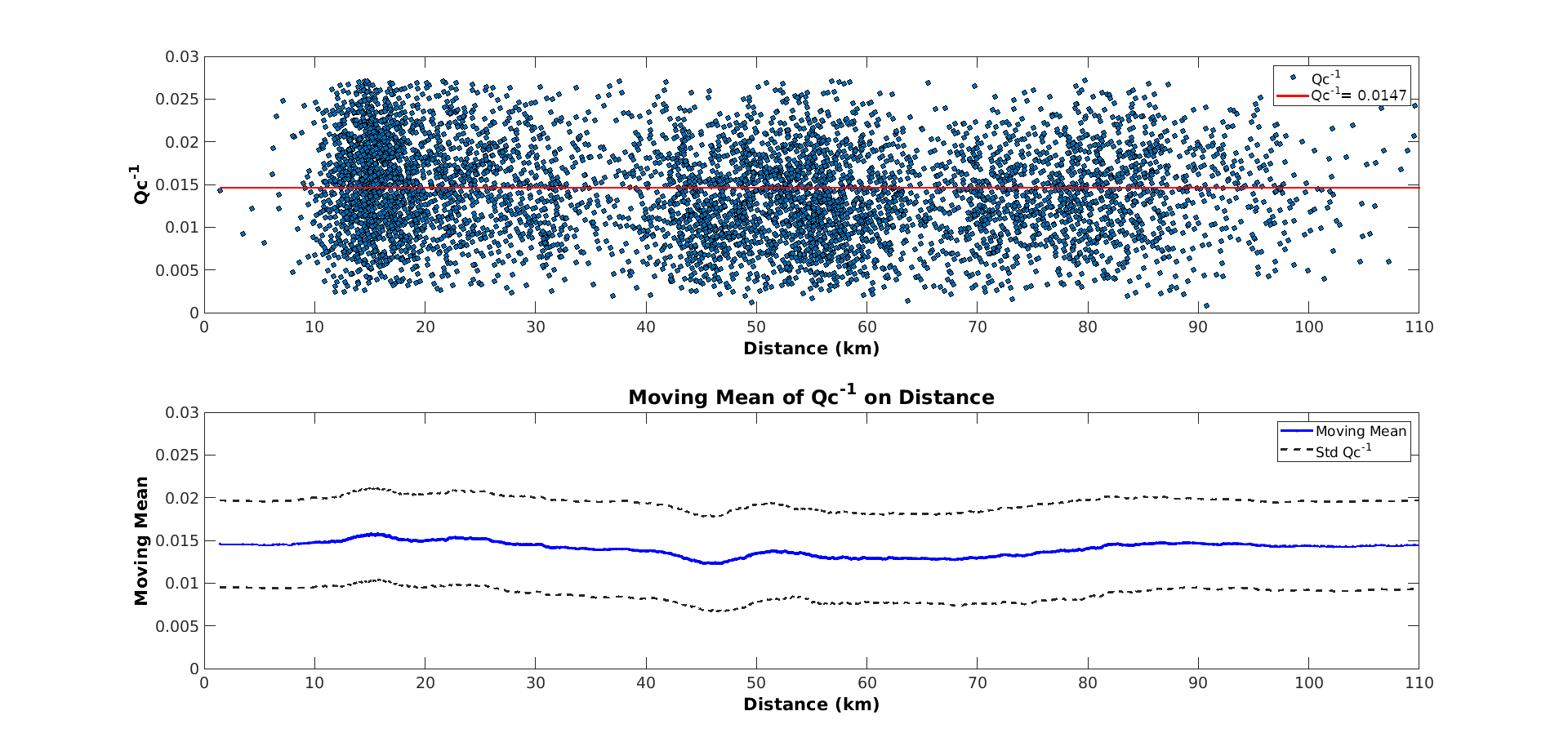


Figure S5. Upper panel: the ${Q_{c}}^{-1}$ measurements obtained for each waveform (light blue circles) as a function of the ray length within a central frequency of 1.5 Hz. The average value (horizontal red line) within the selected ray length range is 0.014. Lower panel: moving average (blue line) and standard deviation (black dashed lines) computed on Qc-1 measurements.


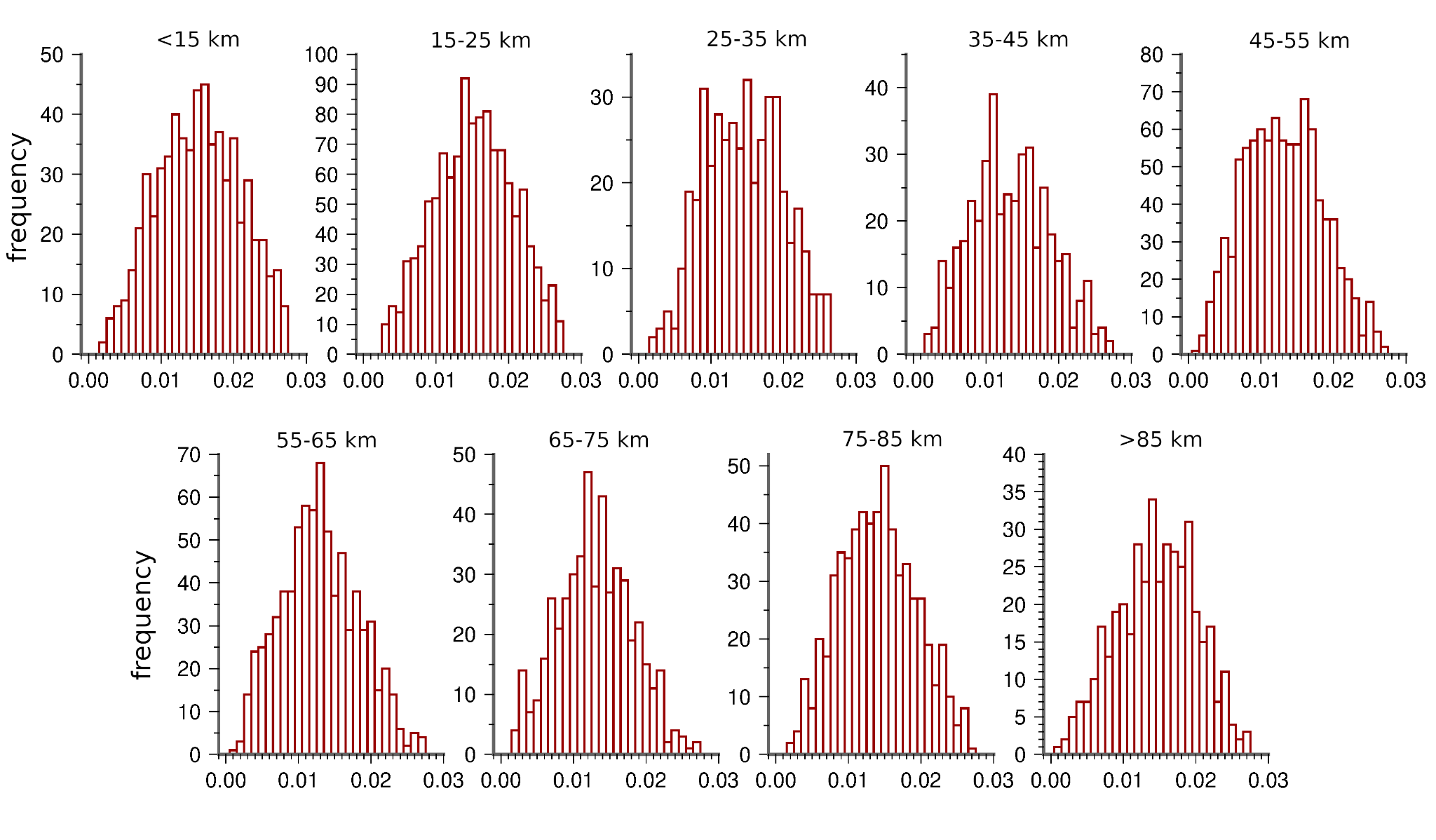


Fig S6. Qc ^-1^ histograms at different windows of hypocentral distances (shown on the top of each panel).

Table S1. Qc^-1^ mean value and standard deviation at 9 hypocentral distance ranges, assessing the stability of Qc^-1^ values at all hypocentral distances.

| Hypocentral distance range (km) | $Qc^{-1} \pm\Delta Qc^{-1}$ |
| --- | --- |
| < 15 | 0.015 $\pm$ 0.006 |
| 15 - 25 | 0.015 $\pm$ 0.005 |
| 25 - 35 | 0.015 $\pm$ 0.005 |
| 35 - 45 | 0.013 $\pm$ 0.006 |
| 45 - 55 | 0.013 $\pm$ 0.005 |
| 55 - 65 | 0.013 $\pm$ 0.005 |
| 65 - 75 | 0.013 $\pm$ 0.005 |
| 75 - 85 | 0.014 $\pm$ 0.005 |
| > 85 | 0.015 $\pm$ 0.005 |


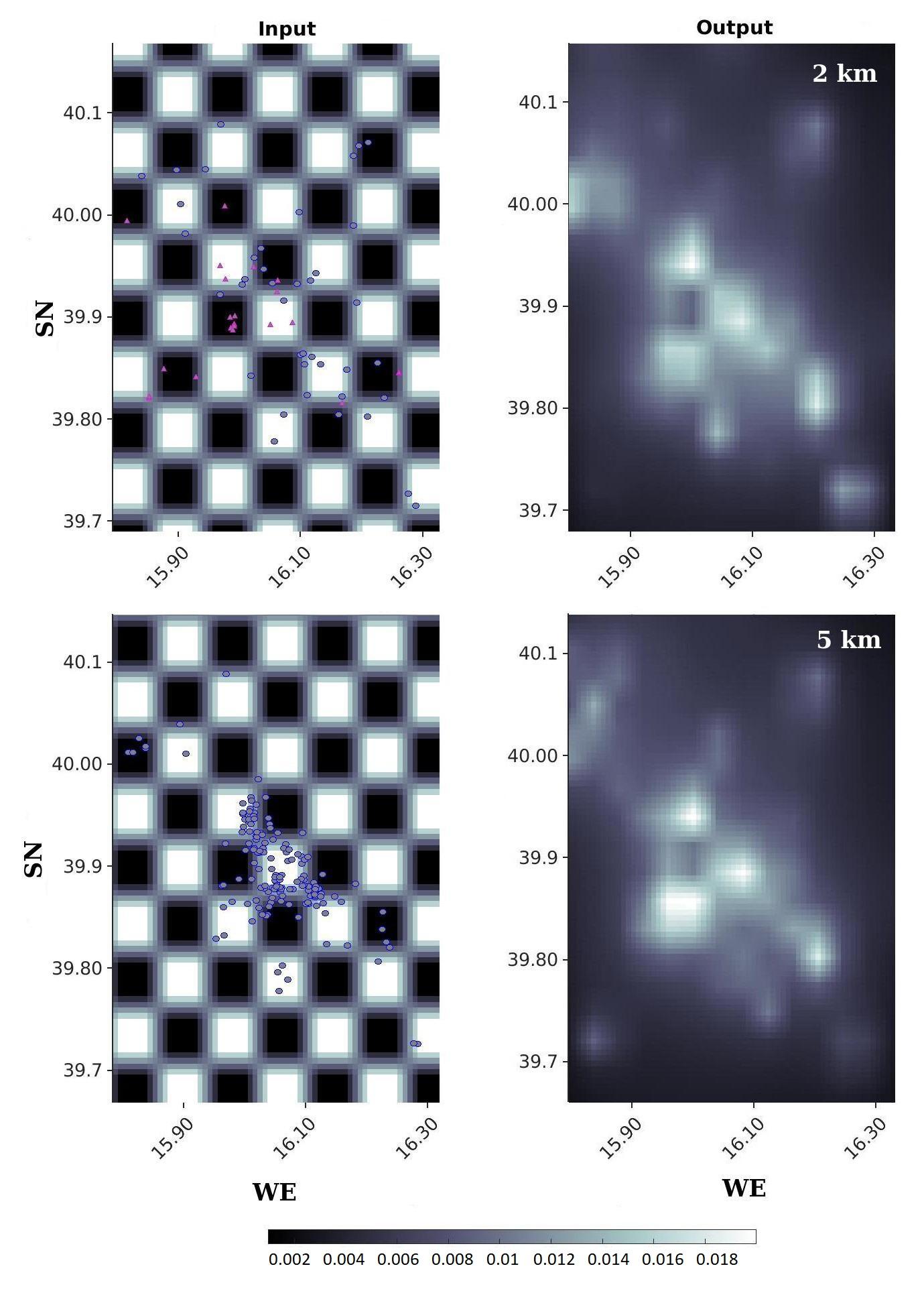


Figure S7. Checkerboard tests (input on the left side and output on the right side) for the Qc-1 anomaly at 2 and 5 km depth.


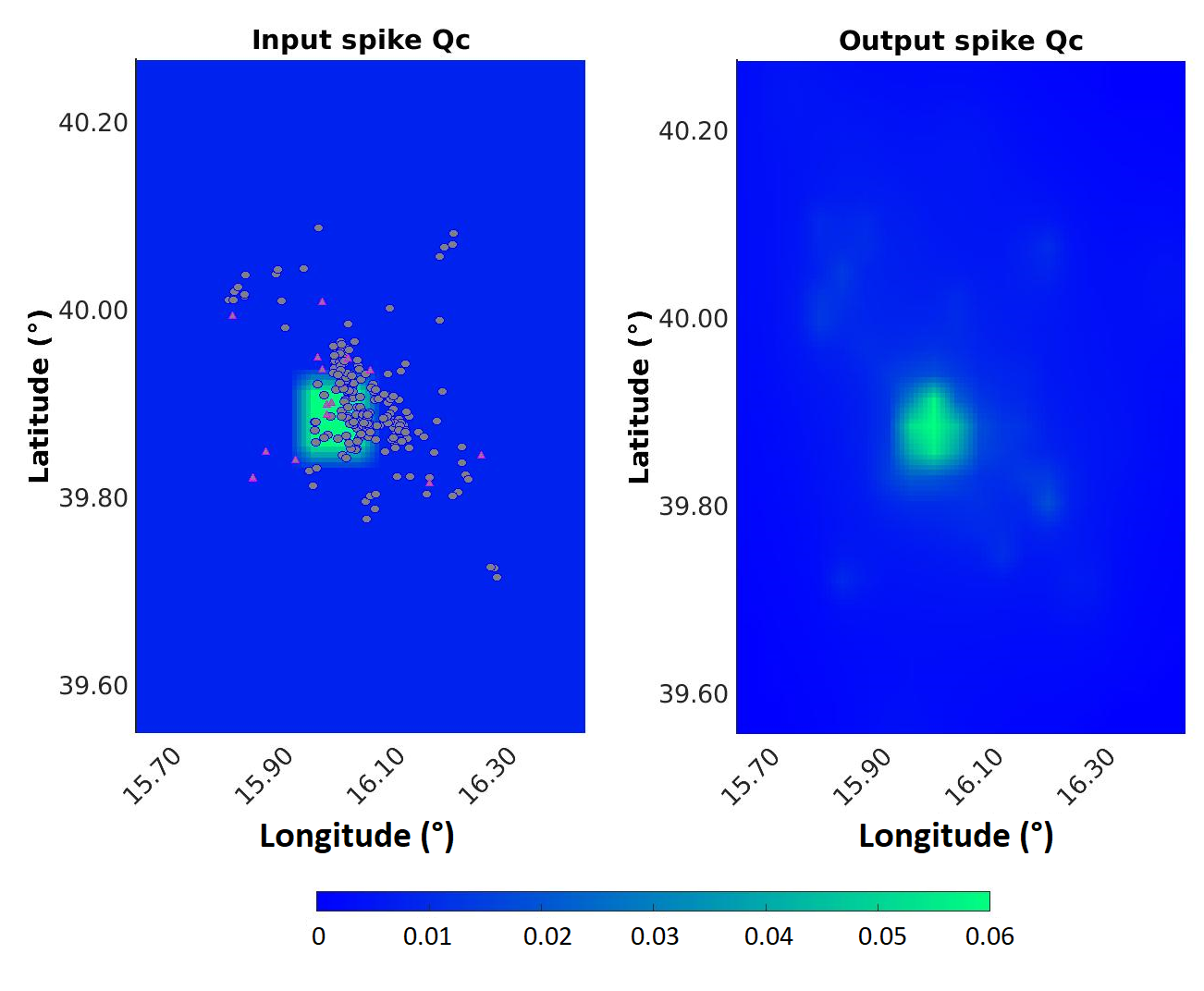


Figure S8. Spike tests at 5 km depth of the high absorption anomaly shown in Figure 3.


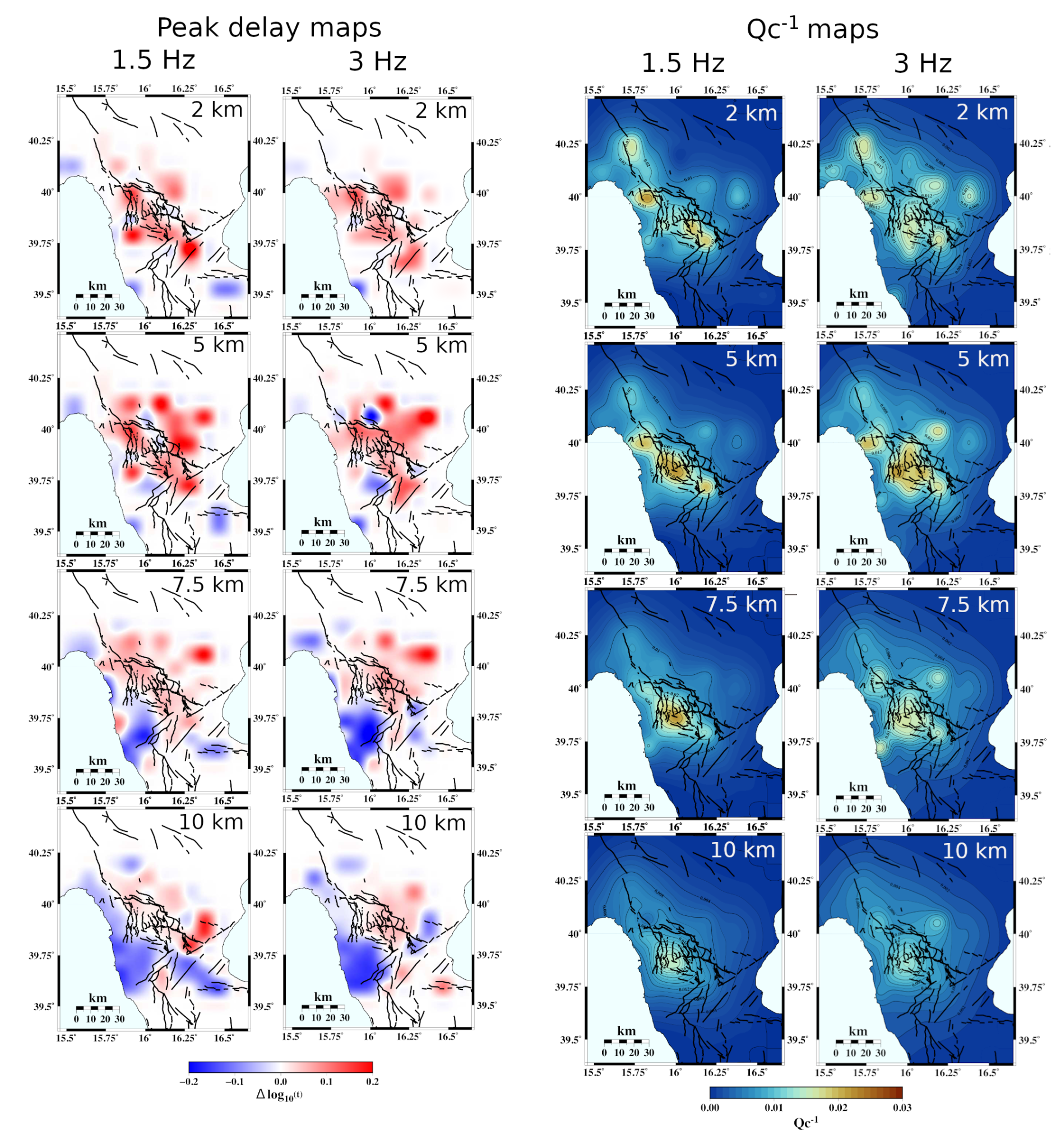


Fig. S9: Qc-1 maps shown at different depths (2 km, 5 km, 7.5 km, and 10 km) computed using two central frequencies: 1.5 Hz, discussed in the main text, and 3 Hz, shown for comparison.
